# Targeting tumor-associated macrophage: an adjuvant strategy for lung cancer therapy

**DOI:** 10.3389/fimmu.2023.1274547

**Published:** 2023-11-13

**Authors:** Lei Liu, Genwang Chen, Sisi Gong, Rongfu Huang, Chunmei Fan

**Affiliations:** Department of Laboratory Medicine, Second Affiliated Hospital of Fujian Medical University, Quanzhou, China

**Keywords:** tumor-associated macrophages, combined therapy, immunotherapy, lung cancer, PD-1/PD-L1

## Abstract

The emergence of immunotherapy has revolutionized the treatment landscape for various types of cancer. Nevertheless, lung cancer remains one of the leading causes of cancer-related mortality worldwide due to the development of resistance in most patients. As one of the most abundant groups of immune cells in the tumor microenvironment (TME), tumor-associated macrophages (TAMs) play crucial and complex roles in the development of lung cancer, including the regulation of immunosuppressive TME remodeling, metabolic reprogramming, neoangiogenesis, metastasis, and promotion of tumoral neurogenesis. Hence, relevant strategies for lung cancer therapy, such as inhibition of macrophage recruitment, TAM reprograming, depletion of TAMs, and engineering of TAMs for drug delivery, have been developed. Based on the satisfactory treatment effect of TAM-targeted therapy, recent studies also investigated its synergistic effect with current therapies for lung cancer, including immunotherapy, radiotherapy, chemotherapy, anti-epidermal growth factor receptor (anti-EGFR) treatment, or photodynamic therapy. Thus, in this article, we summarized the key mechanisms of TAMs contributing to lung cancer progression and elaborated on the novel therapeutic strategies against TAMs. We also discussed the therapeutic potential of TAM targeting as adjuvant therapy in the current treatment of lung cancer, particularly highlighting the TAM-centered strategies for improving the efficacy of anti-programmed cell death-1/programmed cell death-ligand 1 (anti-PD-1/PD-L1) treatment.

## 1 Introduction

Lung cancer remains the leading cause of cancer-related death, with approximately 1.8 million deaths reported in 2020 worldwide (1). Moreover, lung cancer is the second most commonly diagnosed cancer, with approximately 2.2 million newly diagnosed cases. This represents approximately 11.4% of diagnosed cases of cancer and 18.0% of deaths globally (1). In the United States of America, the incidence of advanced lung cancer has declined precipitously owing to the implementation of annual screening through low-dose computed tomography. However, the rates of localized disease suddenly increased by 4.5% per annum (2). Due to earlier advances in detection and treatment, such as improved staging and video-assisted thoracic surgery, the overall mortality rate of patients with lung cancer has markedly declined in recent years. However, the 5-year survival rate of patients with lung cancer following diagnosis between 2010–2014 in most countries remains only 10–20% (3).

Thus, there is a pressing need to identify novel therapeutic targets and prognostic predictors for the treatment of lung cancer. Apart from conventional operative treatment and chemotherapy/radiotherapy, targeted agents have been extensively studied and successfully applied to the care of patients, particularly targeting mutations in the epidermal growth factor receptor (EGFR). Numerous drugs targeting EGFR, including dacomitinib, erlotinib, gefitinib, and afatinib, have been approved by the US Food and Drug Administration (FDA). The effectiveness of EGFR tyrosine kinase inhibitors (TKIs) in prolonging the overall survival and progression-free survival of patients with lung cancer has been thoroughly investigated (4). In addition, immune checkpoint molecule blockade therapy is currently emerging as a standard treatment for lung cancer. Programmed cell death-1 (PD-1) and its ligand (PD-L1) are two key “brakes” in the field of immunotherapies. Importantly, monoclonal antibodies (e.g., atezolizumab, pembrolizumab, and nivolumab) have been produced and utilized in clinical use. Despite the success of current PD-1/PD-L1 antibodies in treating lung cancer, some patients who exhibit initial but not sustained response continue to be associated with poor outcomes (5). In addition, despite impressive response rates, therapeutic resistance inevitably arises following both targeted therapy and immunotherapy (6). The mechanisms of acquired and primary resistance to molecular therapy are multi-factorial, and the lack of adequate preclinical immune models and insufficient available clinical data limit our present understanding of the precise mechanisms at play (7). Thus, new treatment options and a more precise selection of medications are required to extend the long-term survival of patients with lung cancer. In this review, we expounded on the advancements in immunotherapy targeting PD-1/PD-L1, and discussed its potential application to the treatment of lung cancer with a focus on the tumor microenvironment (TME).

Cancer cells are embedded within the TME, which is composed of the extracellular matrix and multiple types of stromal cells, including fibroblasts, immune cells, and vascular networks (8). Tumor cell–TME interactions are complex and bidirectional, affecting tumor progression at multiple levels (9). Accumulating evidence indicates that oncogenic mutations impact angiogenesis and are associated with accumulation of immune cells and the phenotype in the TME. These effects are exerted through an increase in the secretion of chemokines and cytokines by tumor cells (10). Tumor-associated macrophages (TAMs) are the most prominent non-cancerous cells associated with tumor progression, which play important roles in contributing to carcinogenesis, neoangiogenesis, neurogenesis, remodeling of the immunosuppressive TME, recurrence, chemoresistance, and metastasis (11–13). As a result, TAM reprogramming by pharmacological strategies has garnered considerable research attention over the past few years (12). TAMs affect tumor development in numerous manners; however, the specific mechanisms underlying these effects remain unclear, thereby hindering the pharmacological use of TAMs in clinical practice. The aim of this review was to expound on the roles of TAMs in lung cancer, including TAMs and TAM-associated molecules or particles. Moreover, the mechanisms underlying the effects of TAMs on tumorigenesis were categorized and discussed. Furthermore, based on our enhanced understanding of TAM biology, targeting TAMs has become a promising strategy for anti-cancer treatments. Finally, strategies for modifying TAMs as anti-cancer agents were also explained in this review.

An immunosuppressive TME is the key mechanism underlying the development of resistance to immune checkpoint inhibitors (ICIs) (14). TAMs play a major role in tumor immune evasion. Altering immune responses is crucial to successful ICI therapy, and targeting TAMs is an important strategy for combating tumor resistance (15). Furthermore, TAM residence has been shown to repress the anti-tumor efficacy of anti-PD-1/PD-L1 agents through different effects (16). Thus, in addition to describing the role of TAMs in regulating the tumorigenesis of lung cancer and focusing on emerging TAM-tailored strategies, we also discussed the potential therapeutic effectiveness of TAM-targeted therapy as adjuvant treatment in combination with targeted therapies, immunotherapies, and other treatment strategies. Among the combination therapies, we emphasized the correlation between TAMs and PD-1/PD-L1 expression and highlighted TAMs as a potential therapeutic target for overcoming resistance to anti-PD-1/PD-L1 agents.

## 2 TAMs contribute to lung cancer development

**Figure 1:**
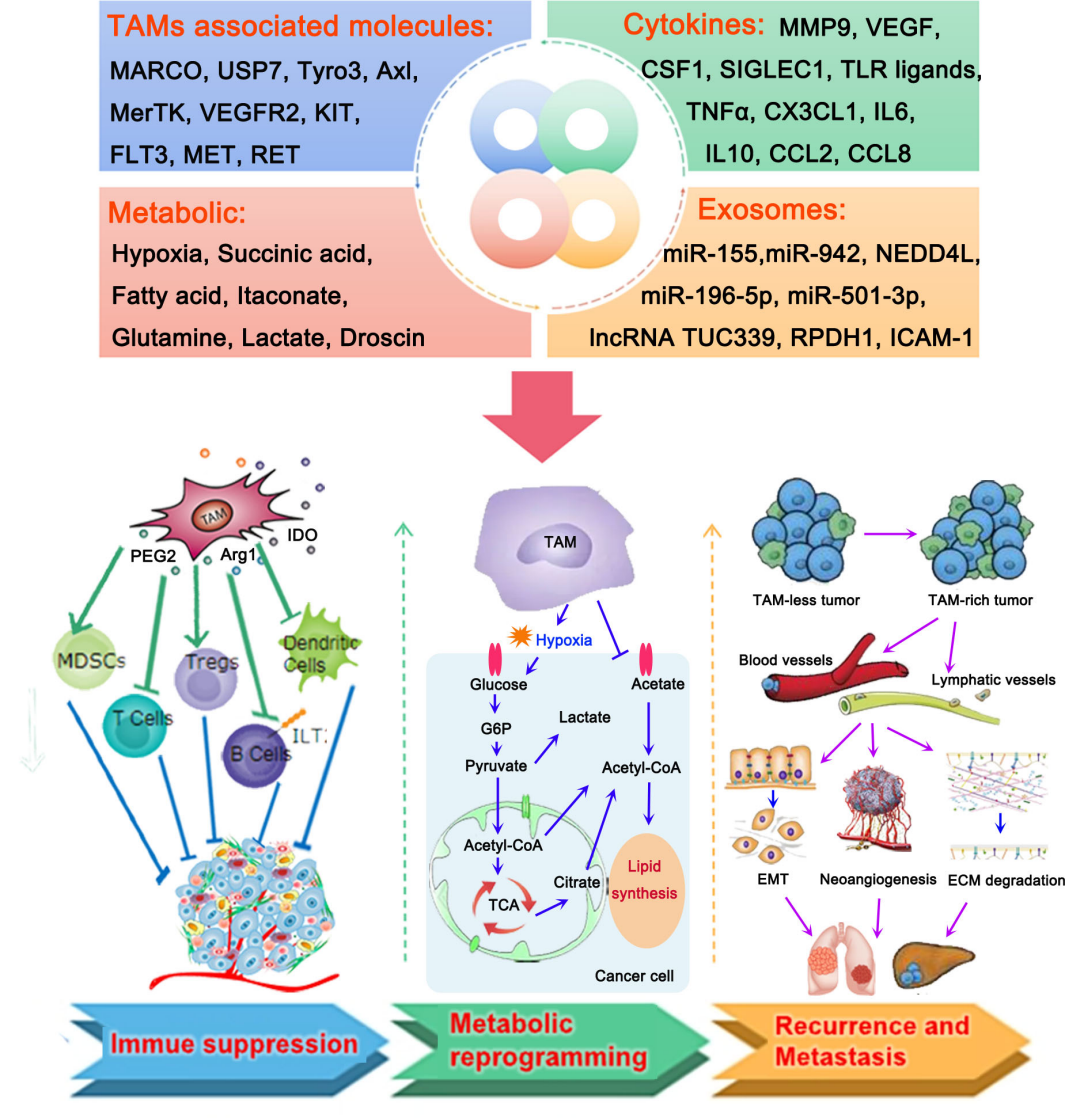
Roles of tumor-associated macrophages (TAMs) in the microenvironment of lung cancer. TAMs, TAM-associated molecules, cytokines, metabolic, and exosomes produced by TAMs play important roles in the development of lung cancer by different mechanisms, including the formation of an immunosuppressive tumor microenvironment (TME) and metabolic reprogramming, thereby contributing to the recurrence and metastasis of lung cancer. TAMs contribute to the suppressive TME by activating myeloid-derived suppressor cells (MDSCs) and regulatory T cells (Tregs), and inactivating T cells, B cells, and dendritic cells. They mainly participate in the processes of glycol metabolism and lipid metabolism to regulate the metabolic reprogramming of lung cancer cells. Accumulation of TAMs in lung cancer could promote the migration of cancer cells to blood vessels and lymphatic vessels. This is followed by the induction of epithelial-to-mesenchymal transition (EMT) and neoangiogenesis, as well as the degradation of the extracellular matrix, thus resulting in the recurrence and metastasis of lung cancer.

Over the past decade, phagocytes, most of which are derived from bone marrow monocytes, have been recognized as an important part of innate immunity (17). These cells can be polarized into different statuses in response to different microenvironments and stimulating factors. According to the different activation states, functions, and secretion of cytokines, macrophages can be typically divided into two groups, namely M1 macrophages (pro-inflammatory) and M2 macrophages (anti-inflammatory) (18). It has been reported that M1-like polarization is activated by granulocyte-macrophage colony-stimulating factor (GM-CSF), lipopolysaccharides, and interferon-γ (IFN-γ) (18). Activation of the M1-like pathway is accompanied by a release of cytokines, including tumor necrosis factor α (TNF-α) and interleukin 1β (IL-1β), and IL-6, which facilitates a pro-inflammatory response in defense against pathogenic insults and malignant tumor cells (19). In contrast, the M2 phenotype is activated by IL-4 and IL-13; the ability of these cells for endocytosis is enhanced by their high expression of CD206 (20). M2-like macrophages may be involved in immune regulation, angiogenesis, and the promotion of T helper 2 cell differentiation and tumor progression by secreting transforming growth factor β (TGFβ), IL-10, and other anti-inflammatory cytokines (21). Macrophages that have infiltrated into the TME are termed TAMs. TAMs retain the pro- and/or anti-inflammatory functions of macrophages. Thus, the roles of TAMs in the TME are complex and changeable. Considering that TAMs are emerging as key modulators of tumorigenesis, it is necessary to characterize their biological functions and critical mechanisms involved in facilitating cancer cell extravasation and intravasation (**Figure 1**).

### 2.1 TAMs regulate the immune cell response in lung cancer

An immunosuppressive TME acts as a decisive factor in cancer dissemination, immune evasion, and the establishment of a suppressed immune microenvironment. Immune cells are the main components of the immune system, controlling the balance between suppressor and cytotoxic responses in lung cancer (22). Data showed that TAMs could hinder the functions of other immune cells, particularly natural killer cells and T lymphocytes, and facilitate the formation of the immune-suppressive microenvironment. TAMs promote T cell exhaustion by expressing the transcription factor interferon regulatory factor 8 (IRF8) (23). TAMs or TAM-related molecules, such as IL37 receptor (IL37R), macrophage receptors with collagenous structure (MARCO), CD200, and c-Maf, inhibit TAM proliferation and cytokine production, and block the activation of cytotoxic T cells and natural killer cells. Notably, targeting these molecules with an antibody or knockout of these molecules may repolarize TAMs, thereby recovering the anti-tumoral ability of T cells and natural killer cells, as well as the activities of downmodulated regulatory T cells (24–26).

In addition, a recent study indicated that the induction of fibrosis led to numerically and functionally impaired dendritic cells and altered TAM phenotypes. These effects likely contribute to increased lung cancer progression, impaired T cell immune surveillance, and failure of ICI efficacy (27). Taking into account the immunosuppressive TME caused by TAMs, reprogramming TAMs to modulate the anti-tumor immune responses has been considered a novel therapeutic approach for lung cancer therapy (28). For example, hydroxychloroquine (HCQ) could promote the change of M2 type to M1 type TAMs, exerting anti-non-small cell lung cancer (anti-NSCLC) cell effects and resulting in CD8+ T cell infiltration into the TME (29). However, of note, some research studies demonstrated that the function of TAMs as primarily immunosuppressive cells may not be fully applicable to patients with early-stage lung cancer. For example, tumor-associated monocyte/macrophage lineage cells in early-stage human lung tumors consist of a mixture of classical tissue monocytes and TAMs. Singhal et al. found that it was classical monocytes, rather than TAMs, that predominantly suppress the responses of T cells (30). These findings may explain the limited response of some patients to immunotherapy.

### 2.2 TAMs affect the metabolic reprogramming of lung cancer

Metabolic reprogramming of the cancer and immune cells is a putative driver of the anti-cancer immune responses in cancer. TAMs associated with the metabolic profile of the immune microenvironment have a prominent effect on tumor progression (31). It was recently found that macrophages are more able to consume glucose and produce lactate. This contradicts the traditional notion that tumor cells generate lactate via glycolysis (32). It has also been reported that TAMs affect the glycolysis of tumor cells by secreting TNF-α. Simultaneously, TAM depletion using clodronate failed to abrogate aerobic glycolysis, thus improving tumor response to anti-cancer treatment (33). A recent study revealed that the metabolism of methionine is a crucial mediator of immune evasion and tumor initiation. The investigators also pointed out the correlation between methionine metabolism and TAMs, and proposed a key role of methionine metabolism in TME modelling (34). Moreover, it has been shown that the depletion of cholesterol induces tumor-promoting effects in TAM. Another study revealed that cholesterol efflux transporters are upregulated in TAMs involved in cholesterol biosynthesis and metabolism compared with alveolar macrophages. Data showed an opposing regulation of cholesterol homeostasis in lung tumor tissue versus TAMs, suggesting a new anti-tumor strategy based on cholesterol depletion (35). Thus far, few studies have investigated heme catabolism in TAMs. Nonetheless, scholars identified a distinct subset of TAMs, accompanied by elevated levels of heme oxygenase-1 (HO-1). This subset plays an essential role in shaping a pro-metastatic TME promoting epithelial-to-mesenchymal transition (EMT), angiogenesis, and immunosuppression. These TAMs were identified as a novel anti-metastatic target and blood prognostic biomarker in lung cancer (36). Nevertheless, additional animal and clinical studies are needed to further verify their clinical value.

### 2.3 TAMs contribute to lung cancer neoangiogenesis, lymphangiogenesis, and EMT

Neoangiogenesis, lymphangiogenesis, and EMT are three biological processes that occur before metastasis ultimately takes place. Metastasis is one of the most common causes of cancer-related mortality. Recent studies have suggested that other factors, apart from the malignant behavior of tumor cells, determine the occurrence of distant metastasis. It has been shown that stromal cells, particularly TAMs, act as major promoters of these processes (37–39). For instance, Hwang et al. found that TAMs are significantly associated with both lymphangiogenesis and angiogenesis, contributing to disease progression in 349 patients with NSCLC. In addition, similar to a high combined M2 ratio, a high M2 ratio was recognized as a strong biomarker of unfavorable prognosis in NSCLC (40). Zheng et al. showed that changes in the spatial density and distribution of TAMs between the invasive margin and the tumor center could predict patient survival in NSCLC (41). Mechanistically, numerous research studies have reported an essential role of TAMs in regulating EMT formation, leading to upregulated levels of mesenchymal markers, while attenuating those of epithelial markers (42–45). The polarization of TAMs can also be regulated by these signal molecules, including mitogen-activated protein kinase (MAPK) family molecules, phosphatidylinositol-4,5-bisphosphate 3-kinase (PI3K), JUN N-terminal kinase/signal transducer and activator of transcription 3 (JNK/STAT3), Wnt-β, etc. (46–49). Besides, TAMs can trigger PI3K/AKT signaling to bypass pro-apoptotic cytokines, such as tumor necrosis factor-related apoptosis-inducing ligand (TRAIL), as well as other chemokines that direct tumor cell localization to the premetastatic niche (50). Therefore, TAMs play an important role in promoting the migration of cancer cells from the pre-invasive site to the metastatic lesions.

Based on extensive data, the molecular regulation of TAMs in tumor progression is crucial for the further development of cancer-targeted strategies. Advances in anti-tumor drugs and anti-cancer action mechanisms have been recently achieved (51). An immunosuppressive TME is an important factor in tumor epigenetics, immune evasion, dissemination, and differentiation. TAMs play essential roles in the TME and are, therefore, of critical importance in cancer therapy. For instance, M2 polarization of TAMs can promote resistance to some classical chemotherapy drugs, such as cisplatin (52), gefitinib (53), and imatinib (54), via the secretion of cytokines, including TGFβ, IL-8, epidermal growth factor (EGF), and TNF-α. It has been reported that targeting of the immune-responsive gene 1 (IRG1) reverses the immunosuppressive function of TAMs and enhances cancer immunotherapy (55).

In addition, a genetic decrease of sphingosine-1-phosphate receptor 1 (S1PR1) or cytochrome P450 family 4 subfamily A (CYP4A) in TAMs could reduce the formation of the pre-metastatic niche in lungs and reduce the metastatic burden. This is accompanied by the polarization of TAMs polarizing away from the M2 type, thus rendering S1PR1 and CYP4A potential therapeutic targets in lung cancer (56, 57). Apart from TAM-related molecules, researchers also found that lactate (a metabolite derived from macrophages) acts as a key factor in maintaining cancer growth through activation of mechanistic target of rapamycin complex 1 (mTORC1). Subsequently, it was shown that lactate restrains the expression of vacuolar ATPase subunit mediated by transcription factor EB (TFEB) (58). In light of these encouraging preclinical findings, the use of TAM-targeting strategies holds promise for future clinical application.

### 2.4 TAMs may promote tumoral neurogenesis in lung cancer

Nerves are a key constituent of the TME that can promote tumor growth, invasion, and metastasis. Tumor cells release neuropeptides or neurotrophic factors to promote axonogenesis and result in tumor innervation (59). Neurotransmitters regulate both pro-immunity and anti-immunity responses, also influencing the TME through various mechanisms (12). Thus, potential therapeutic alternatives could be developed by arresting abnormal tumor neurogenesis and disrupting cancer cell–neuron communication. Immunological nerves and mediators play pivotal roles in body homeostasis by interacting with each other through a variety of mechanisms (60). Moreover, evidence revealed a direct signaling pathway from lesion-specific activated monocytes to spinal progenitor cells, promoting regenerative neurogenesis in zebrafish (61). While the interplay between neurons and macrophages has been explored, studies chiefly focused on neurodegenerative diseases (62) or ischemic events (63). Thus far, few studies have focused on the interaction between TAMs and tumor innervation. In 2022, Tang et al. discovered a direct mechanism through which TAMs promote *de novo* neurogenesis via a subset exhibiting neuronal phenotypes and expression of pain receptors related to cancer-directed nocifensive behaviors. Through elucidation of the TAM transcriptome dynamics, they found a “macrophage-to-neuron-like cell transition” (MNT) phenomenon that directly promotes tumor neurogenesis. This evidence was derived from fate mapping in lung carcinoma models and macrophage depletion studies. The investigators also recognized macrophage-specific SMAD3 as a key modulator of MNT promotion, which represents a precision therapeutic target for cancer-related pain (64). As one of the most important parts of the TME, nerves communicate with immune cells and cancer cells in complex ways. Additional international research in this field is warranted to provide a novel and promising scientific basis for improvement in medical care.

### 2.5 TAM-derived exosomes affect lung cancer progression

Recent evidence has demonstrated that exosomal signaling is strongly correlated with tumor growth (65), metastasis (66), immune regulation (67), and drug resistance (68), particularly for exosomes derived from macrophages. Wang et al. found that TAM-derived exosomes were able to promote chemoresistance and aerobic glycolysis in lung cancer through the stabilization of c-Myc by inhibiting NEDD4 like E3 ubiquitin protein ligase (NEDD4L) (69). In addition to metastasis, exosomes derived from M2 macrophages also contribute to the progression of lung cancer by numerous functional miRNAs, such as miR-942, miR-155, miR-196-5p, and miR-501-3p (70–72). Taken together, the available evidence indicates that targeting TAM-derived exosomal signaling represents a promising and novel strategy for improving the treatment and management of lung cancer.

### 2.6 TAM subtypes in the TME of lung cancer

Classical TAMs, also termed M2 macrophages, have characteristic biomarkers, including CD301, CD115, CD206, CD204, and CD163. Biomarkers of M1 macrophage polarization include CD80 and CD86. These molecules form the basis for identification of TAMs. Early in the process of oncogenesis, TAMs adopt an M1-like pro-inflammatory phenotype and initiate an immune response that terminates the formation of tumors. As tumors progress, the M2-like functional transformation of TAMs is triggered by different mechanisms, thereby directing them to participate in angiogenesis, immunosuppression, and other tumor-supporting processes (73). The heterogeneity and plasticity of TAMs presents a potential therapeutic target directed against reversal of the TAM phenotype in the tumor. Understanding the mechanism underlying the switch of the TAM phenotype is essential for application to clinical care. As reported in the literature, TAM phenotypic differentiation is mainly controlled by cytokines (74). Recent studies reported that nutrients, which can be consumed, stored, recycled, or converted to signaling molecules, are crucial regulators of macrophage responses in tumors (75). The competition between tumor cells and macrophages leads to a limited supply of nutrients (e.g., glucose, lipids, and amino acids) to immune cells, which affects the differentiation and function of TAMs (76). Thus, remodeling of the TME that induces changes in macrophage nutrient uptake and polarization status is a feasible strategy for enhancing the anti-tumor immunity and resistance to oxidative stress, as well as suppress immune escape. A limited oxygen supply in the developing tumors also induces the M2-like functional transformation of TAMs by means of direct effects, metabolic influence, lactic acidosis, angiogenesis, remodeled stroma. Subsequently, oxygen deprivation urges TAMs to participate in immunosuppression, angiogenesis, and other tumor-supporting processes (77). Based on these data, a hypoxic TME may be a crucial regulator of TAM functional transformation.

In addition to the typical subtypes of TAMs, some atypical TAM types occupying only a very small proportion but displaying vital functions were found by bulk and single-cell transcriptomic analysis. For example, Zhang et al. discovered a triggering receptor expressed on myeloid cells 2 (TREM2)-positive (+) TAM subtype in lung cancer that could stratify patients depending on their responses to immunotherapy. TREM2+ TAMs were observed with various types of anti-inflammatory cytokines, displaying an immunosuppressed phenotype similar to that of M2. Moreover, these TAMs potentiated T cell dysfunction, including increased differentiation to forkhead box P3-positive (FOXP3+) regulatory T cells and impaired anti-tumor activity of CD8+ T cells. These effects facilitated immune evasion of NSCLC (78).

Iron-loaded TAMs, which possess anti-tumorigenic activity, may be a promising prognostic biomarker for patients with lung cancer (79). Garrido-Martin et al. found a particular subtype of TAMs in human lung cancer. This subtype was similar to M2 macrophages, but co-expressed a strong/hot M1-like signature (M1^hot^ TAMs). M1^hot^ TAMs were associated with high numbers of tumor-infiltrating CD8+ tissue-resident memory T cells and improved outcomes (80). Metastasis-associated macrophages are a type of TAMs present at the metastatic site. A study revealed that loss of caveolin 1 (CAV1) in metastasis-associated macrophages could drive metastatic growth in the lung via enhanced angiogenesis (81). These atypical TAM types demonstrate a distinct role in tumorigenesis compared with classical TAMs. Nevertheless, the results of a functional trial revealed the clinical significance of these TAMs as prognostic and predictive biomarkers or as therapeutic targets for anti-cancer therapy.

## 3 Novel therapeutic strategies against TAMs in lung cancer

**Figure 2:**
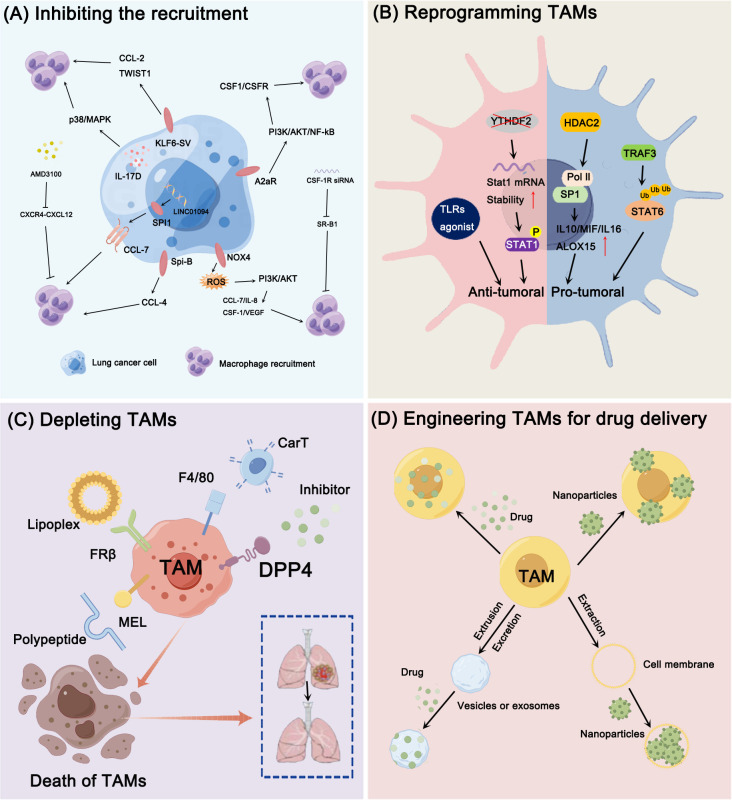
Engineering TAMs for lung cancer therapy. **(A)** Signals produced by lung cancer cells regulate the recruitment of macrophages. Interleukin (IL)-17D, Adenosine 2a receptor (A2aR), NADPH oxidase 4 (NOX4), Spi-B, Krüppel like factor 6 splice variant 1 (KLF6-SV1), and CCL7 are key regulators to play the tumor-promoting roles by inducing macrophages infiltration and recruitment through multiple signal pathways. And agents such as CSF-1R antibodies and CXCL12 inhibitors could hind the recruitment of TAMs and significantly inhibit the cancer metastasis. **(B)** Newly identified molecules that directly regulate the M2/M1 switching of TAMs. YTHDF2 deficiency facilitated the reprogramming of TAMs by targeting interferon-γ-STAT1 signaling; TRAF3 promoted M2 polarization via regulating STAT6 K450 ubiquitination, HDAC2 could regulate the M2-like TAM phenotype via acetylation of histone H3 and transcription factor SP1. **(C)** Depletion of TAMs could improve the prognosis of lung cancer. Using the folate-modified lipoplex, CAR T cells targeting F4/80, hybrid peptide composed of melittin (MEL) or DPP4 inhibitor are effective strategies to lead to cell death of TAMs. **(D)** Graphical illustrations of engineered TAM for drug delivery. TAMs, TAM-derived exosomes, TAM-membrane-coated NPs are identified as drug deliveries for lung cancer therapy.

TAMs play vital roles in restricting the ability of the immune system to combat cancer and hinder the anti-cancer efficacy of most therapies currently used in clinical practice. Consequently, TAMs are considered a potentially powerful target for future immunotherapies against cancer (**Figure 2**). In this section, we summarized novel strategies against TAMs and provided promising pharmacological approaches for lung cancer therapy (**Table 1**).

**Table 1:** Therapeutic approaches for TAMs reprogramming in lung cancer.

Target or agent	Object	Mechanisms and effects	Ref.
Hydroxychloroquine (HCQ)	Lung cancer cell lines and mice	HCQ can promoting the change of M2 type to M1 type TAMs, exerting anti-non-small cell lung cancer cell effects and resulting in CD8+ T cell infiltration into the TME	(29)
Clodronate	Lung cancer cell lines and mice	Depletion of TAM by clodronate was sufficient to abrogate aerobic glycolysis and tumor hypoxia, thereby improving tumor response to anticancer therapies.	(33)
Immune-responsive gene 1 (IRG1)	Mice	IRG1 expressed in TAMs can reverse the immunosuppressive function of TAMs and enhance the efficacy of anti-PD-L1 immunotherapy	(55)
Lactate	Mice	Lactate inhibits ATP6V0d2 expression in tumor-associated macrophages to promote HIF-2α-mediated tumor progression	(58)
SMAD3	Mice	SMAD3 as a key modulator of “macrophage-to-neuron-like cell transition” promotion, which directly promoting tumor neurogenesis, representing a precision therapeutic target for cancer-related pain.	(64)
M2-macrophage-derived exosomes (MDE)	Lung cancer cell lines and mice	MDE were able to promote chemoresistance and aerobic glycolysis in lung cancer through the stabilization of c-Myc by inhibiting NEDD4L.	(69)
Colony stimulating factor 1 receptor (CSF1R)	Lung cancer cell lines and mice	CSF1R inhibition reduced the tumor growth rate and TAM density, as well as increased sensitivity to chemotherapy.	(82, 83)
Emactuzumab (anti-CSF1R monoclonal antibody)	Patients received immunotherapy (NCT02323191)	Emactuzumab in combination with atezolizumab treatment resulted in considerable overall response rate in patients with NSCLC previously treated with ICI, accompanied by increased rates of fatigue and skin rash, compared with typical atezolizumab alone.	(84)
N6-methyladenosine reader YTH N6-methyladenosine RNA binding protein F2 (YTHDF2)	Lung cancer cell lines and mice	YTHDF2 deficiency in TAMs suppress tumor growth and enhance cancer immunotherapy by reprogramming TAMs toward an antitumoral phenotype and increasing their antigen cross-presentation ability, which in turn enhanced CD8+T cell-mediated antitumor immunity.	(85)
Imidazoquinoline	Mice	Intravenous injection of imidazoquinoline lead to a prominent decrease in tumor growth, along with repolarization of TAMs to a pro-inflammatory type and enhancement of anti-tumor T cell responses.	(86)
Dihydroartemisinin	Lung cancer cell lines and mice	Dihydroartemisinin-triggered ferroptosis of TAMs lead to DNA damage and activation of the NF-κB signaling pathway to reprogram TAMs to an M1-like type, thus providing a new approach for lung cancer therapy.	(87)
Marsdenia tenacissima	Lung cancer cell lines and mice	Marsdenia tenacissima extract can disturb the cell–cell interaction between cancer cells and TAMs by regulating HDGF and increasing the polarization of macrophages from the M2 type to the M1 type.	(88)
A hybrid peptide composed of melittin and the pro-apoptotic peptide d2 (MEL-dKLA)	Lung cancer cell lines and mice	MEL-dKLA selectively leads to cell death of M2 macrophages without affecting other immune cells, resulting in lower tumor weights and angiogenesis.	(89)
Folate receptor β (FRβ)	Mice	Treatment of a folate-modified liposome significantly inhibit tumor growth *in vivo* by inducing tumor cell and macrophage apoptosis, reducing tumor proliferation, and inhibiting tumor angiogenesis.	(90)
Chimeric antigens receptor-T cells targeting F4/80 (F4.CAR-T)	Mice	F4.CAR-T cells can successfully infiltrate tumor lesions and delay tumor growth in a manner comparable to that observed with PD-1 blockade by killing macrophages.	(91)
Dipeptidyl peptidase 4 (DPP4)	Mice	DPP4 inhibitor reduces TAMs and enhances the anti-PD-L1 effect by inhibiting the expression of NOX1 and NOX2.	(92)
A. annua-derived nanovesicles	Mice	The nanovesicles can remold TAMs for reprogramming anti-cancer immunity and facilitating tumor eradication by inducing the cyclic GMP-AMP synthase/stimulator of interferon genes (cGAS/STING) pathway.	(93)
Microfluidics-enabled nanovesicle delivers CD47/PD-L1 antibodies (NCPA)	Mice	NCPA can enhance the anti-tumor efficacy and reduced immunotoxicity in lung cancer by promoting the remodeling of TAMs to an anti-tumoral state.	(94)
Engineered immunocytokine PD-1-IL2v	Mice	The use of engineered immunocytokine PD-1-IL2v in combination with anti-PD-L1 overcomes tumor immunity resistance and improves therapeutic efficacy both by reprogramming immunosuppressive TAMs and enhancing T cell receptor immune repertoire diversity.	(95)
Immunoglobulin-like transcript 4 (ILT4)	Lung cancer cell lines and mice	Inhibition of ILT4 can prevent TAM-mediated immunosuppression and improve the efficacy of treatment with a PD-L1 inhibitor in NSCLC patients with EGFR mutation.	(96)
Nanoparticles based on calcium bisphosphonate (CaBP-PEG)	Lung cancer cell lines and mice	These nanoparticles efficient *in vivo* TAM depletion, and imaging-guided radioisotope-enhanced cancer therapy.	(97)
PLX, an inhibitor of CSF1R	Mice	The combination of PLX with systemically administered cisplatin provided additional benefits, causing a significant decrease in tumor burden versus monotherapy with PLX or cisplatin by regulating differentiation and recruitment of monocytes.	(98)

ATP6V0d2, macrophage-specific vacuolar ATPase subunit; NEDD4L, NEDD4 like E3 ubiquitin protein ligase; HIF-2α, Hypoxia-inducible factor-2α; HDGF, heparin binding growth factor; NOX1, nicotinamide adenine dinucleotide phosphate oxidase 1; NOX2, nicotinamide adenine dinucleotide phosphate oxidase 2; NF-κB, nuclear factor-κB; NSCLC, non-small cell lung cancer; STING, stimulator of interferon genes; TAM, tumor-associated macrophage.

### 3.1 Inhibition of macrophage recruitment

Colony stimulating factor 1/colony stimulating factor 1 receptor (CSF1/CSF1R), C-C motif chemokine ligand 2/C-C motif chemokine receptor 2 (CCL2/CCR2), CCL5, and vascular endothelial growth factor (VEGF) are classic regulatory factors that affect monocyte recruitment to tumors and shape their function within the TME (99). Therapeutic strategies targeting those molecules have also utilized monoclonal antibodies or receptor antagonists. For example, several preclinical studies have shown that CSF1R inhibition reduced the tumor growth rate and TAM density, as well as increased sensitivity to chemotherapy (82, 83). Rong et al. developed siRNA targeting CCR2, delivered by cationic nanoparticles (NPs). The siRNA showed conspicuous targeting of macrophages in the bone marrow, human peripheral blood, and spleen (100). Apart from those chemokines, macrophages are also recruited and established by signals produced by tumor cells in a tumor-promoting mode. It has been demonstrated that IL-17D promotes lung cancer tumorigenesis by inducing TAM infiltration through the MAPK signaling pathway (101). Adenosine 2a receptor (A2aR) is positively correlated with TAM infiltration by promoting chemokine secretion and factor polarization via activation of the PI3K/AKT/nuclear factor-κB (PI3K/AKT/NF-κB) signal pathway (102). It was recently found that NADPH oxidase 4 (NOX4), Spi-B, Krüppel like factor 6 splice variant 1 (KLF6-SV1), and CCL7 are highly expressed in lung cancer cells. These factors play tumor-promoting roles by inducing macrophage infiltration and recruitment through multiple signaling pathways (74, 103–105). To hinder the recruitment of TAMs, agents such as CSF1R antibodies and C-X-C motif chemokine ligand 12 (CXCL12) inhibitors, have been developed; these agents significantly inhibit cancer metastasis (98, 106). However, a phase 1b study (NCT02323191) assessed the anti-cancer activity, safety, pharmacodynamics, and pharmacokinetics of the anti-CSF1R monoclonal antibody emactuzumab in combination with atezolizumab in patients with advanced cancer who received immunotherapy. The combination treatment resulted in considerable overall response rate in patients with NSCLC previously treated with ICI, accompanied by increased rates of fatigue and skin rash, compared with typical atezolizumab alone (84). The interactions among components of the TME are intricate. The interruption of macrophage recruitment might lead to the activation of other processes promoting tumor progression. Hence, more studies are required to investigate the mechanisms underlying the signal transmission between cancer cells and the human system.

### 3.2 Reprogramming of TAMs

Recently, numerous strategies were developed to facilitate the M2/M1 switching of TAMs. Some of those have demonstrated remarkable clinical efficacy with low toxicity by regulating TAM polarization. Ma et al. found that N6-methyladenosine reader YTH N6-methyladenosine RNA binding protein F2 (YTHDF2) deficiency facilitated the reprogramming of TAMs by targeting IFN-γ/STAT1 signaling. This suggested that YTHDF2 inhibition is an effective approach to enhancing cancer immunotherapy (85). A diphtheria toxin−based fusion toxins that target human EGFR was recently generated and found to activated a proinflammatory phenotype of TAM (107). And another recombinant fusion protein containing interleukin-3 fused to truncated diphtheria toxin, called Tagraxofusp, was also reported to have promising therapeutic response rate by reprograming TAM polarization (108). Anfray et al. reported that toll-like receptor (TLR) agonists, such as poly(I:C), imiquimod, or resiquimod, were able to polarize macrophages toward M1-like anti-tumor effectors *in vivo* and *in vitro* (109). Intravenous injection of imidazoquinoline, another TLR agonist, which site-specifically and quantitatively coupled to the nanobodies of the macrophage mannose receptor (MMR) on TAMs, led to a prominent decrease in tumor growth, along with repolarization of TAMs to a pro-inflammatory type and enhancement of anti-tumor T cell responses (86). Recently, it was shown that TNF receptor associated factor 3 (TRAF3) plays a vital role in M2 polarization by regulating STAT6 K450 ubiquitination in macrophages (110). In addition, histone deacetylase 2 (HDAC2) could regulate the M2-like TAM phenotype via acetylation of histone H3 and transcription factor SP1 (111). Despite the emergence of TAM reprogramming as an effective strategy for immunotherapy, most approaches were unable to reprogram TAMs *in situ* due to their low efficacy, non-specificity, or potential side effects. In the latest study, Zhang et al. generated exosomes with the clustered regularly interspaced short palindromic repeat interference (CRISPRi) internal engineering and the TAM-specific peptide external engineering on the exosome membrane. This approach enabled selective homing to tumor tissue and induced TAM polarization to the M1 type (112). This engineered exosome succeeded in reprogramming TAMs *in situ* and inspired researchers for future work.

Immunometabolism in the tumor microenvironment has been a research hot spot in recent years. Numerous metabolites have been identified to regulate the biological functions of TAMs (113). It was reported that glucose metabolism promotes the differentiation of macrophages towards tumor promotion. Lactate, a metabolic waste product at the terminal of the glycolysis process, is regarded as an active signaling molecule that exerts control over the polarization and function of TAMs via receptor-mediated signaling pathways (114). And immunometabolic therapies targeting glycolysis and lactate are still being found to play their anti-tumor roles (114). Support for amino acid metabolism is also required for the pro-cancer effect of TAMs. It was reported that methionine adenosyltransferase II alpha (MAT2A) induces TAM polarization by upregulating the expression of S-adenosylmethionine via the methylation of histones (34). Besides, glutamine metabolism inhibition has been shown to reprogram TAMs towards a proinflammatory phenotype. Intratumoral high potassium (K+) has shown immune-suppressive potency in T cells. Researchers found that the inwardly rectifying K+ channel Kir2.1 deficiency could disturb the electrochemically-dependent glutamine uptake, engendering TAM metabolic reprogramming from oxidative phosphorylation toward glycolysis, and sequentially boosting local anti-tumor immunity (115). Additionally, CD40 signaling could rewire fatty acid and glutamine metabolism to stimulate the anti-tumorigenic functions of macrophages (116).

Many studies have shown that tumor cells depend even more on anti-phagocytosis molecules, also called phagocytosis checkpoints, to evade immune surveilliance (117, 118). Therefore, identification and intervention with phagocytosis checkpoints might provide a new approach for restoring the phagocytic capacity of TAMs to eliminate tumor cells. For example, macrophage phagocytosis was restored after treatment with CD47 antibodies, and this macrophage-mediated phagocytosis was further enhanced in the presence of chemotherapeutic drugs (119).

Numerous studies suggested that natural compounds may affect the human immune system by targeting TAMs to enhance the immune TME in lung cancer and display a natural anti-tumor effect. For example, a study reported that dihydroartemisinin-triggered ferroptosis of TAMs led to DNA damage and activation of the NF-κB signaling pathway to reprogram TAMs to an M1-like type, thus providing a new approach for lung cancer therapy (87). Furthermore, Fu et al. found that *Marsdenia tenacissima* extract could disturb the cell–cell interaction between cancer cells and TAMs by regulating heparin binding growth factor (HDGF) and increasing the polarization of macrophages from the M2 type to the M1 type (88). Natural compounds (e.g., flavonoids, polyphenols, quinones, and saponins) are able to regulate multiple targets related to tumors and involve multiple mechanisms (120). It has been shown that multiple natural compounds reduce the toxicity of chemotherapy and radiotherapy in patients with lung cancer (121). Hence, the use of natural compounds has become a novel strategy for the adjuvant treatment of cancer. Focusing on the exploration of new natural products targeting TAMs can be a promising direction for future research.

### 3.3 Depletion of TAMs

In addition to the reprogramming of TAMs to a more active phenotype, some agents can selectively deplete TAMs from the TME and enhance their anti-tumor activity. For example, Lee et al. targeted M2-type TAMs using a hybrid peptide composed of melittin (which preferentially binds to M2-type TAMs) and the pro-apoptotic peptide d2 (which induces mitochondrial apoptosis upon infiltration of the cell membrane). It is possible that the compound selectively leads to cell death of M2 macrophages without affecting other immune cells, resulting in lower tumor weights and angiogenesis (89). Folate receptor β (FRβ) is highly expressed in TAMs and, thus, has become a promising target of TAM-based cancer therapy. A folate-modified lipoplex was designed and injected into models of lung cancer models. The administration resulted in the depletion of FRβ-positive macrophages and inhibition of tumor growth (90, 122).

Other strategies for the depletion of macrophages in the TME have also been developed. For example, Sánchez-Paulete et al. used chimeric antigens receptor-T cells targeting F4/80 (F4.CAR-T), a recognized macrophage marker, to kill macrophages (91). The results showed that F4.CAR-T cells were able to successfully infiltrate tumor lesions and delay tumor growth in a manner comparable to that observed with PD-1 blockade. This evidence supported the use of CAR-T cells targeting TAMs as a strategy for enhancing anti-tumor effectiveness. It was reported that dipeptidyl peptidase 4 (DPP4) inhibitor reduces TAMs and enhances the anti-PD-L1 effect by inhibiting the expression of NOX1 and NOX2 (92). A recent study revealed that endothelial cells and macrophages are the major types of senescent cells in KRAS-driven murine lung cancer. The data also showed that clearance of senescent macrophages significantly decreased the cancer burden and prolonged survival (123). This high-quality research highlighted that targeting the depletion of TAMs can be a potential therapeutic avenue and strategy for the prevention of cancer.

### 3.4 Engineering TAMs for drug delivery

Apart from their direct reprogramming, TAMs can also be engineered to acquire tumor-tropic and migratory properties for drug delivery in the treatment of lung cancer. One such approach involves the construction of drug-loaded macrophages through incubation with drugs. For example, Qiang et al. produced a biomimetic delivery system by loading doxorubicin into a cell line resembling a murine macrophage (RAW264.7). Biological experiments indicated that the drug encapsulated in macrophages could efficiently convert absorbed near-infrared light into thermal energy, resulting in a rapid release of doxorubicin (124).

Additionally, it has been reported that photosensitizer chlorin e6 is carried by TAMs and exhibits an anti-cancer effect via photodynamic reprogramming. These findings provided a new direction for combining photodynamic therapy (PDT) with anti-tumor immunotherapy (125). Considering the toxicity and drug-loading ratio, most TAM-based delivery systems utilize macrophages as carriers directly loading anti-tumor agents, where they charge NPs containing drugs rather than loading the drugs directly. The nanovesicle is the most common type of polymer NPs. Plant-derived nanovesicles have been mentioned as the main mechanism for interactions between kingdoms. A recent study found that medicinal plant-derived mitochondrial DNA remolded TAMs for reprogramming anti-cancer immunity and facilitating tumor eradication by inducing the cyclic GMP-AMP synthase/stimulator of interferon genes (cGAS/STING) pathway via nanovesicles (93). Moreover, the development of dual-targeted nanofabricated dandelion that targets TAMs exhibited effective anti-tumor activities and reduced adverse effects *in vivo* in mice with A549 tumors (126). In another study, a microfluidic-based nanovesicle was developed using a CD47/PD-L1 antibody. Use of this nanovesicle enhanced the anti-tumor efficacy and reduced immunotoxicity in lung cancer by promoting the remodeling of TAMs to an anti-tumoral state (94). In addition to NPs, drug-loaded microbubbles or exosomes can also be used as drug carriers targeting TAMs. For example, imiquimod (R837)- and docetaxel-loaded microbubbles were designed in a recent investigation to remold the TME through polarization of M2-phenotype TAMs to M1-phenotype TAMs (127). Moreover, M1-macrophage-derived exosomes exhibited the ability to deliver a variety of anti-tumor drugs. Kim et al. utilized M1-macrophage-derived exosomes to load paclitaxel using a nano-formulation and sonication method. This approach was effective in the treatment of drug-resistant tumors (128). Collectively, the evidence above suggests that the engineering of macrophages to regulate the immunosuppressed TME and application of M1 macrophages as drug transporters may be promising strategies in cancer therapy.

## 4 Targeting TAMs to address anti-PD-1/PD-L1 resistance in lung cancer

Clinical trials have investigated the effectiveness of anti-PD-1/PD-L1 treatment in lung cancer. The results showed that, compared with chemotherapy, treatment with pembrolizumab, nivolumab, or atezolizumab led to marked improvement in overall and progression-free survival (129). Thus, the development of PD-1/PD-L1 inhibitors has been a great advancement in the treatment of lung cancer, providing new therapeutic options and improved outcomes for patients with advanced disease. While PD-1/PD-L1 antibodies have presented significant promise in lung cancer therapy, some deficiencies associated with this type of treatment remain. For example, only a proportion of patients exhibit a good response to treatment with anti-PD-1/PD-L1 agents. Only approximately 20% of patients with cancer respond to immunotherapy, and poor responses may restrict therapeutic efficacy and result in local recurrences and/or distant metastases (130). Moreover, in some patients who exhibit an initial but not sustained response, tumors may develop resistance to PD-1/PD-L1 inhibitors over time, leading to disease progression (131). Hence, ongoing research or new investigations on anti-PD-1/PD-L1 agents are necessary to improve the efficacy of treatment and reduce the adverse events associated with this type of therapy. The identification of markers that could help predict patients who most likely would benefit from therapy is another strategy for improving patient outcomes.

### 4.1 Advancement of PD-1/PD-L1 treatment in lung cancer

Treatments blocking the PD-1/PD-L1 axis have been linked to promising clinical outcomes in a substantial number of patients with advanced cancer. However, the response rate to anti-PD1/PD-L1 monotherapy is low, not exceeding 8% (132). To reduce the presence of other immunosuppressive mechanisms in the TME and the chance of non-response due to insufficient PD-L1 expression in most tumors, new PD-1/PD-L1 inhibitors or new methods based on anti-PD-1/PD-L1 treatment are applied to clinical use (**Table 2**). Given the low response rate, preclinical and clinical studies assessing combination therapies are currently ongoing to improve clinical outcomes (141). Clinical trials focusing on the combination of PD-1/PD-L-1 inhibitors with other chemotherapy agents are emerging (**Table 2**). The use of nanomaterials could enhance treatment efficacy by directly delivering the drugs to the affected area. Hence, NP-based immunotherapy has become a hot topic in this field. The Antigen Release Agent and Checkpoint Inhibitor (ARAC) is a NP co-delivering an anti-PD-L1 agent and volasertib (a polo like kinase 1 [PLK1] inhibitor). The latest research in a model of metastatic lung cancer revealed that the use of ARAC led to a five-fold reduction in the doses of anti-PD-L1 agent and volasertib. In addition, ARAC has shown efficacy in another model of lung cancer (KLN-205), which is unresponsive to combination treatment with cytotoxic T-lymphocyte associated protein 4 (CTLA4) and PD-1 antibodies (142). Combining ICI with epigenetic modifiers is another approach to improving the clinical outcome. This combination regimen arises from evidence indicating that targeting epigenetic elements may enhance anti-tumor immunity through remodeling of the TME.

**Table 2:** Ongoing clinical trials of emerging anti-PD-1/PD-L1 drugs for the treatment of lung cancer.

Drug	Description	Trial identification number	Phase	Design	Ref.
Cemiplimab	Anti-PD-1 mAb	NCT03409614	III	Combination with platinum in 466 patients with stage III/IV NSCLC in the absence of ROS1, ALK, or EGFR genomic tumor aberrations	(133)
HER-2 B-cell peptide vaccines	Vaccines were engineered to represent the trastuzumab- and pertuzumab-binding sites (anti-PD-1)	NCT01417546	I	Patients with metastatic, incurable solid tumor malignancy	(134)
Tiragolumab plus atezolizumab	Anti-PD-1 mAb	NCT03563716	II	Patients with chemotherapy-naïve, recurrent, or metastatic PD-L1-positive disease, without alterations in EGFR or ALK were recruited from 41 clinics across the USA, Asia, and Europe	(135)
Sintilimab plus nab-paclitaxel and carboplatin	Combination of PD-1 inhibitor sintilimab with chemotherapy	NCT04326153	II	Patients with NSCLC (stage IIIA/IIIB) who were treated with sintilimab plus nab-paclitaxel and carboplatin for 2–3 cycles prior after neoadjuvant treatment	(136)
GEMSTONE-302	Sugemalimab (a PD-L1 inhibitor) plus chemotherapy	NCT03789604	III	Patients with squamous (stage IV) or non-squamous NSCLC without known EGFR sensitizing mutations, RET, ROS1, or ALK fusions	(137)
Durvalumab plus tremelimumab	Anti-PD-1 and CTLA4 mAb	NCT02888743	II	Patients with metastatic NSCLC that developed during previous treatment with PD-L1 were randomized (1:1:1) to receive either durvalumab plus tremelimumab alone or with either low-dose or hypo-fractionated radiotherapy	(138)
Atezolizumab	PD-L1 inhibitor	NCT03285763	III/IV	619 patients with NSCLC (stage IIIB/IV) who experienced disease progression after chemotherapy were enrolled to assess the safety and efficacy of atezolizumab monotherapy	(139)
Toripalimab	Anti-PD-1 mAb	NCT03301688	I	41 patients with advanced NSCLC who received at least three lines of therapy	(140)

ALK, anaplastic lymphoma kinase; CTLA4, cytotoxic T-lymphocyte associated protein 4; EGFR, epidermal growth factor receptor; mAb, monoclonal antibody; nab, nanoparticle albumin-bound; NSCLC, non-small cell lung cancer; PD-1, programmed cell death-1; PD-L1, programmed cell death-ligand 1; ROS1, ROS proto-oncogene 1.

Cancer vaccines offer an effective approach to augmenting cancer-specific immune responses by redeploying the immune system. Tumor cells stimulate anti-tumor immunity through the use of tumor antigens, which could be administered as cells, proteins, nucleic acids, etc. (143). Cell-based vaccines are the primary form of cancer vaccines; of note, dendritic cells have shown significant results in clinical trials (144). PD-L1, an important tumor-associated antigen, plays a key role in immunotherapy and immunoprevention. Therefore, through this method, PD-L1 can be effectively imported into dendritic cells for antigen presentation. For example, Zeng et al. (145) generated a specific fragment of mesothelin (MSLN) combined with the peptide immunogen PD-L1 and GM-CSF (MSLN-PD-L1-GMCSF) based on the new anti-PD-L1 vaccine approach. Guo et al. (146) synthesized a single B-cell vaccine (PD-1-Vaxx), and tested its anti-tumor properties and immunogenicity in the syngeneic BALB/c mouse model. Besides, Maxime Thoreau et al. demonstrated that cooperation between T cells and macrophages is required to achieve the effects of a therapeutic vaccine (147). And a listeria-based tumor vaccine was reported to benefit anti-PD-1 therapy by skewing macrophage polarization (148) Adoptive cell transfers have emerged as a disruptive approach to treat disease. C Wyatt Shields 4th et al. reported an engineered particle referred to as a “backpack” that can robustly adhere to macrophage surfaces and regulate cellular phenotypes *in vivo*. Backpacks evade phagocytosis for several days and release cytokines to continuously guide the polarization of macrophages toward antitumor phenotypes, resulting in reduced metastatic burdens and slowed tumor growths (149).

Additionally, a phase I first-in-human study (NCT01417546) was conducted to assess the optimal dose of immunologic/biological agents, as well as the antigenicity when combining two peptide vaccines with B cell epitopes engineered to provide binding sites for trastuzumab and pertuzumab. The results demonstrated that this vaccine exhibits strong anti-cancer activity (134). Although therapeutic cancer vaccines attracted considerable attention in the last decade, the majority of cancer vaccines remain at the preclinical and clinical research stages. Thus, there is a need for more specific antigens and platforms for vaccine development.

Single-agent immunotherapy has gained widespread acceptance as a first-line therapy for lung cancer with high PD-L1 expression in tumors. Nevertheless, most patients respond poorly to this type of therapy, and the mechanisms of resistance remain unclear (150). At present, there are considerable challenges to further clinical applications of PD-1/PD-L1 blockade therapy, such as the lack of predictive and prognostic markers, the small fraction of patients that benefit from this treatment, adverse events related to treatment, and acquired resistance. PD-L1 expression is the most widely used predictive marker for anti-PD-1/PD-L1 therapy. However, this biomarker is not definitive, as patients with low or absent PD-L1 expression can still respond to therapy (151). Hence, further investigation for the identification of biomarkers is warranted to maximize the utilization of ICIs in advanced lung cancer and improve cancer immunotherapy. In **Table 3**, we list several novel biomarkers that can predict a favorable response and prolong survival after PD-1 axis blockade therapy for lung cancer. Insights into tumor- and host-specific immune factors that can inform both prognostication and prediction of response permit the recognition of effective biomarkers for immunotherapy. Nonetheless, further research is required for the discovery of cost-effective markers with high sensitivity and specificity.

**Table 3:** Novel biomarkers that can predict a favorable response after PD-1 axis blockade therapy for lung cancer.

Biomarker	Description	Cohort	Multivariate HR	AUC	Ref.
CD44	CD44	128 patients with NSCLC who received single-agent PD-1 axis inhibitors	PFS: 0.31 (95% CI: 0.11– 0.87) OS: 0.29 (95% CI: 0.09–0.97)	N/A	(152)
LDNs	Low-density neutrophils	31 patients with NSCLC treated with pembrolizumab	N/A	0.908	(153)
TCF1^+^ PD-1^+^ TILs	T-cell factor 1^+^ programmed cell death-1^+^ tumor-infiltrating lymphocytes	768 NSCLC patients who received ICI (PD-1 or PD-L1 blockade) therapy	PFS: 0.25 (95% CI: 0.11–0.77) OS: 0.19 (95% CI: 0.1–0.87)	N/A	(154)
FAP	Fibroblast activation protein	135 patients with advanced NSCLC who received PD-1 blockade therapy	PFS: 2.56 (95% CI: 1.69–3.87) OS: 1.57 (95% CI: 0.99–2.48)	N/A	(155)
LDH	Lactate dehydrogenase	A meta-analysis: 3,429 patients with advanced NSCLC treated with PD-1or PD-L1 inhibitors	PFS: 1.02 (95% CI: 1.00–1.04) OS: 1.19 (95% CI: 1.11–1.24)	N/A	(156)
dNLR	Derived neutrophil-to-lymphocyte ratio		PFS: 1.33 (95% CI: 1.16–1.54) OS: 1.55 (95% CI: 1.33–1.80)		
PLR	Platelet-to-lymphocyte ratio	133 patients who received treatment with a PD-1 monoclonal antibody	PFS: 0.781 (95% CI: 0.500–1.221) OS: 0.566 (95% CI: 0.477–1.498)	0.68	(157)
NLR	Neutrophil-to-lymphocyte ratio		PFS:0.201 (95% CI: 0.060–0.670) OS: 0.413 (95% CI: 0.226–0.754)	0.77	
CD163+ cell	CD163+ cell	152 patients with NSCLC receiving ICI treatment	PFS: 0.61 (95% CI: 0.40–0.94) OS: 0.48 (95% CI: 0.28–0.80)	N/A	(158)
SMS	Somatic mutation signature	248 patients with EGFR/ALK-negative NSCLC treated with anti-PD-1	PFS: 4.32 (95% CI: 2.32–8.06) OS: 3.07 (95% CI: 1.71–5.49)	N/A	(159)
KRAS G12C mutation	KRAS G12C mutation	44 patients with advanced or metastatic NSCLC with elevated PD-L1 expression (≥ 50%), treated with first-line immunotherapy	PFS: 0.46 (95% CI: 0.22–0.95)	N/A	(160)
MER4 ERV	MER4 endogenous retrovirus	Two cohorts of patients with NSCLC treated with PD-1/PD-L1 inhibitors. Cohort 1 n = 89; Cohort 2: n = 70	Cohort 1, PFS: 0.30 (95% CI: 0.20–0.70); OS: 0.40 (95% CI: 0.20–0.90) Cohort 2, OS: 0.60 (95% CI: 0.20–1.30); OS: 0.30 (95% CI: 0.10–1.00)	N/A	(161)
FXR	Farnesoid X receptor	149 patients with NSCLC who received anti-PD-1-based chemoimmunotherapy	PFS: 0.552 (95% CI: 0.315–0.967) OS: 0.377 (95% CI: 0.157–0.905)	N/A	(162)
dNLR	Derived neutrophil-to-lymphocyte ratio	221 patients with advanced NSCLC without EGFR mutations or ALK rearrangements and a PD-L1 TPS ≥50%, who received at least one dose of commercial pembrolizumab monotherapy	PFS: 0.47 (95% CI: 0.33–0.67) OS: 0.32 (95% CI: 0.21–0.49)	N/A	(163)

ALK, anaplastic lymphoma kinase; AUC, area under curve; CI, confidence interval; EGFR, epidermal growth factor receptor; HR: hazard ratio; ICI, immune checkpoint inhibitors; N/A, not available; NSCLC, non-small cell lung cancer; OS, overall survival; PD-1, programmed cell death-1; PD-L1, programmed cell death-ligand 1; PFS, progression-free survival; TPS, tumor proportion score.

### 4.2 Correlation between TAMs and PD-1/PD-L1 expression in lung cancer

TAMs and the expression of PD-1/PD-L1 are related in the context of cancer immunology. The impact of PD-1 expression on TAMs has been well established over the past years. Several preclinical studies have shown that high expression of PD-1 on TAMs is associated with decreased immune activity and poorer outcomes in lung cancer (164, 165). Therefore, the regulation of PD-1 expression on TAMs may be a target for therapies aimed at improving the immune response against cancer. In addition, tumor-infiltrating TAMs are thought to be extrinsic regulators of PD-L1 expression in cancer; their expression is related to the clinical response to PD-L1 inhibitors. This emphasized the significance of understanding the role of TAMs in regulating PD-1/PD-L1 expression and the immune response against cancer. Overall, despite these complexities, the role of PD-1 on TAMs is currently under active investigation. A better understanding of the relationship between PD-1/PD-L1 expression and TAMs in cancer immunotherapy may result in the development of more effective treatments in the future.

To examine the possible factors regulating PD-L1 on cancer by the TME, Shima et al. assessed the relationships of PD-L1 expression in tumors and infiltrating immune cell profiles in lung adenocarcinoma using immunohistochemistry. The investigators found that PD-L1 positivity of the tumor was significantly associated with invasion of the stroma, which was accompanied by an increase in TAMs and FOXP3+ regulatory T lymphocytes. Of these cells, TAMs accumulated significantly in the PD-L1-positive areas of carcinoma cells, which exhibited a cancer cell nidus-infiltrating pattern. *In vitro* experiments also revealed that the expression of PD-L1 in lung cancer cells was significantly upregulated by co-culture with M2-type macrophages (166). In addition, Chen et al. reported that CSF1R+ TAMs are the main components modulating the expression of PD-L1 within the immunosuppressive TME (167). These results suggest a critical effect of TAMs on PD-L1 expression in tumors. Considering the limited research conducted thus far on this aspect, further investigation is required to fully understand the connection between TAMs and the expression of PD-L1 in tumors.

It has been demonstrated that TAMs could express both the PD-1 receptor and PD-L1 (168). Previous studies indicated that tumor cells were able to positively regulate PD-L1 expression in TAMs through several signaling pathways, such as the cyclooxygenase 2/microsomal prostaglandin E synthase 1/prostaglandin E synthase 2 (COX2/mPGES1/PGE2) (169), NF-κB (170), and STAT3 (171). It has also been shown that PD-1 expression in TAMs is upregulated over time during the progression of cancer; however, there is limited knowledge regarding the potential mechanisms involved in this process (172). Considering the high expression of PD-1/PD-L1 axis members in TAMs, more attention has been paid recently to the effect of immune checkpoint factors targeting TAM polarization. Overexpression of both PD-1 and PD-L1 in TAMs inhibited M1-like polarization and supported M2-like polarization. Numerous studies have indirectly shed light on the role of PD-L1 expression in stimulating M2 polarization of TAMs. According to Zhang et al. (173), inhibition of PD-L1 in adenocarcinoma of the lung resulted in an increase in M1 markers and a decrease in M2 markers. They also reported that all PD-1+ TAMs have a surface expression pattern similar to that of M2 macrophages, while PD-1−TAMs exhibit an M1-like profile. These findings indicated that PD-1+ TAMs have a unique transcriptional profile compared with PD-1– TAMs (173). Collectively, the above observations imply that PD-1 expression is tightly associated with an M2-like type of TAMs and affects the onset and/or phagocytosis of TAMs.

Data demonstrated that PD-1 expression in TAMs is negatively correlated with phagocytic potency against cancer cells. Moreover, the use of anti-PD-1/PD-L1 agents *in vivo* could enhance the phagocytosis of macrophages and reduce tumor growth (174). Logistic regression analysis in lung adenocarcinoma showed that infiltration by stromal PD-1+ TAMs is an independent predictor of shorter survival. In addition, patients with high numbers of PD-1+ TAMs in the stroma, but low PD-L1 expression in tumor cells, are linked to the shortest survival time (175). Cell culture studies proposed that the activation of STAT3 by cancer cell-derived GM-CSF is responsible for this relationship (176). The above results indicate that, in addition to the impact of PD-1/PD-L1 on TAM polarization, the presence of PD-1+ or PD-L1+ TAMs in the tumor stroma is a potential prognostic factor in lung cancer. Therefore, a greater understanding of PD-1+/PD-L1+ TAMs will be beneficial for the immunotherapy of patients with lung cancer.

### 4.3 New therapeutic approaches to address PD-1/PD-L1 axis resistance via TAM reprogramming

TAMs are the main cells expressing PD-L1, highlighting the importance for escape from immune system surveillance by cancer cells. In a study of esophageal cancer, TAMs expressed PD-L1 more efficiently to suppress T cells and were more closely associated with immunotherapy responses than cancer cells (177). It has been suggested that anti-PD-1 therapies exert direct or indirect effects on TAMs in both mice and humans. Overall, the correlation between TAM polarization and PD-1/PD-L1 expression in TAMs is complicated. However, numerous studies showed that engineering TAMs that target TAM polarization is a powerful strategy for enhancing the efficacy of anti-PD-1/PD-L1 treatment (178, 179). The use of engineered immunocytokine PD-1-IL2v in combination with anti-PD-L1 overcomes tumor immunity resistance and improves therapeutic efficacy both by reprogramming immunosuppressive TAMs and enhancing T cell receptor immune repertoire diversity (95). TAM receptors, including Tyro3, Axl, and MerTK, are members of the receptor tyrosine kinase family. They are necessary regulators of immune system homeostasis, with multiple effects on the immune response (180). These genes are thought to play major roles in resistance to anti-cancer therapies. For instance, activation of these genes may control inflammatory cytokines, such as IFN-α, TNF-α, IL-1β, and IL-6. There is, therefore, a powerful biological rationale for the combination of a TKI of TAMs with an ICI to overcome drug resistance and enhance the clinical responses of patients with NSCLC. For example, initial preclinical testing showed that crizotinib (a multi-targeted TKI) exerts a potent anti-tumor effect in patients with NSCLC (181). Subsequent phase I and II clinical studies identified favorable and rapid responses to therapy in patients with anaplastic lymphoma kinase (ALK)-positive NSCLC. These data contributed to FDA approval of the use of crizotinib for the treatment of patients with ALK-positive metastatic NSCLC (182). Besides, multiple clinical studies of TAM-targeted agents combined with immunotherapy are in progress, such as BMS-813160 (CCR2/5 inhibitor) combined with Nivolumab (NCT04123379), BL-8040 (CXCR4 antagonist) combined with Pembrolizumab (NCT02826486), Pexidartinib (CSF-1R inhibitor) combined with Durvalumab (NCT02777710), and so on. Apart from TKIs, it has been reported that other crucial immunosuppressive molecules promote the malignant behavior of NSCLC by inducing TAM polarization. Chen et al., for example, found that inhibition of the immunoglobulin-like transcript 4 (ILT4) could prevent TAM-mediated immunosuppression and improve the efficacy of treatment with a PD-L1 inhibitor in NSCLC patients with EGFR mutation (96). Leucine-rich repeat-containing G protein-coupled receptor 4 (LGR4) promotes M2 polarization in monocytes via the R-spondin/LGR4/extracellular signal-regulated kinase/STAT3 (RSPO/LGR4/ERK/STAT3) signaling pathway. In addition, interception of RSPO/LGR4 signaling could overcome the drug resistance of Lewis lung carcinoma to treatment with a PD-1 inhibitor and enhance the response to PD-1 immunotherapy (183). The identification of these molecules provides critical switches of TAM polarization; inhibition of those switches could sensitize lung cancer cells to anti-PD-1 treatment.

## 5 Effects of combining TAM-targeted therapy with other treatments for lung cancer

**Figure 3:**
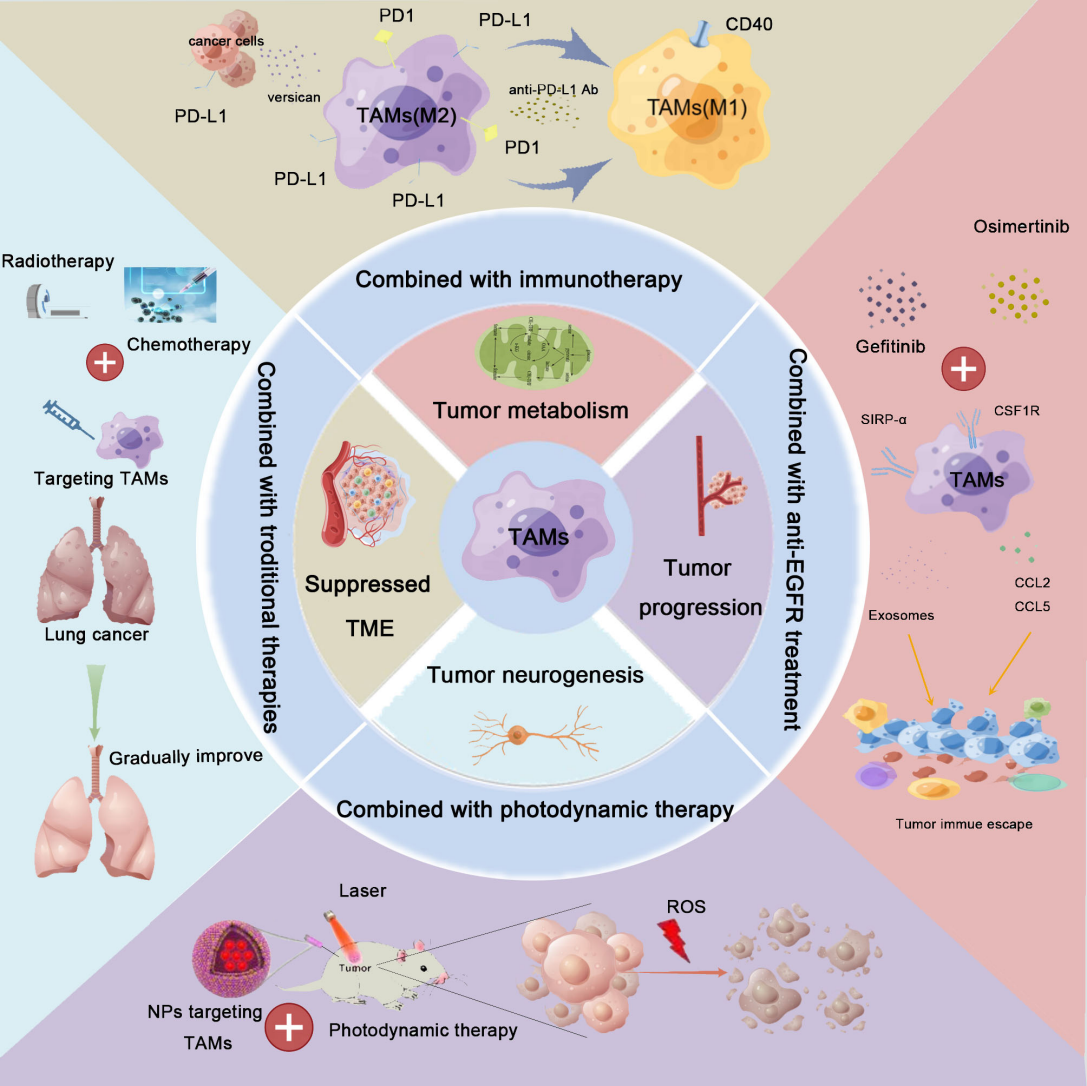
Combination therapy for lung cancer based on TAM-centered strategies. TAMs play crucial roles in lung cancer development, including the regulation of the immunosuppressive tumor microenvironment (TME) remodeling, metabolic reprogramming, neoangiogenesis, metastasis, and promotion of tumoral neurogenesis. Based on these findings, TAM-centered strategies for lung cancer therapy attracted the attention of investigators. Furthermore, the synergistic effects linked to the combination of TAM-targeted therapies with current therapies, including immunotherapy, radiotherapy, chemotherapy, anti-epidermal growth factor receptor (anti-EGFR) treatment, and photodynamic therapy, have also been explored. The combination treatment achieved satisfactory treatment effects.

In the previous section, we elaborated on the combination of TAM-targeted therapy with anti-PD-1/PD-L1 therapy. Apart from immunotherapy, the effects of other treatment modalities (e.g., radiotherapy, chemotherapy, anti-EGFR treatment, or PDT) combined with TAM-targeted therapy have also attracted the attention of researchers (**Figure 3**).

### 5.1 Combination with radiotherapy

Radiotherapy is commonly used for the treatment of numerous types of cancer. However, the occurrence of radio-resistance diminishes the anti-tumor efficacy of X-ray therapy, further restricting its utilization in clinical practice (184). A comprehensive single-cell analysis was performed to explore the mechanisms underlying the development of radio-resistance. The results revealed a substantially reduced fraction of immune cells in irradiated tumors, including depletion of tissue-resident macrophages and infiltration by pro-inflammatory monocytes (185). Another study showed an increase in bone marrow-derived TAMs in irradiated tumors after radiotherapy, which stimulated the resumption of blood flow and facilitated tumor recurrence (186). Therefore, researchers focused on intercepting the aggravation of the immunosuppressive TME caused by the accumulation of M2-type TAMs. For this purpose, TAM-related receptor inhibitors or chemically synthesized multiple nanocomposites were developed to reprogram TAMs or deplete macrophages, which enhanced the efficacy of radiotherapy. For example, TGFβR2 may promote infiltration by T cells in the peripheral blood and within the core of cancer lesions through the upregulation of IFN-β expression in TAMs within irradiated tumors. These results suggested that the use of TGFβR inhibitor in conjunction with radiotherapy may be a new therapeutic approach for the management of lung cancer (187). The chemokine stromal cell-derived factor 1/C-X-C motif chemokine receptor 4 (SDF1/CXCR4) pathway is a crucial mechanism driving the accumulation of TAMs. Therefore, the CXCR4 antagonist plerixafor was used to block this pathway (188). The investigators found that plerixafor could both augment the therapeutic effect of radiotherapy and protect irradiated tissues. Tian et al. discovered a biocompatible nanoplatform based on NPs using radiolabeling chemistry without the use of chelators, efficient *in vivo* TAM depletion, and imaging-guided radioisotope-enhanced cancer therapy (97). CpG-decorated gold NPs were produced to improve the efficacy of radiotherapy by modulating the immune response under exposure to low-dose X-ray irradiation. Gold NPs were used as radio-enhancers to minimize radiation toxicity. Moreover, they acted as nanocarriers for delivering CpG (an agonist of TLR9) to reprogram immunosuppressive M2 TAMs to M1 homologs, thereby priming T cell activation and eliciting innate immunity (189). Based on the above evidence, TAM depletion and repolarization plays a key role in initiating the anti-tumor immune response and overcoming radio-resistance, consequently improving the response of patients with lung cancer to radiotherapy and immunotherapy. Further clinical studies are warranted to explore the pharmacokinetics and pharmacodynamics of the combination strategies.

### 5.2 Combination with chemotherapy

Traditional chemotherapeutic drugs have been discovered and put into clinical use owing to their ability to preferentially kill cancer cells. Nevertheless, it is established that the clinical activity of various chemotherapies is involved in stimulating anti-cancer immunity either by an initial release of immunostimulatory factors from dying tumor cells or through mediation of the off-target effects on immune cells. A better understanding of the specific immunologic mechanisms underlying the efficacy of chemotherapy can assist in identifying superior biomarkers of prognosis and promote the use of synergistic combination therapies that improve clinical efficacy. Combination therapies based on TAMs and chemotherapy have demonstrated considerable synergetic effects in patients with lung cancer. For example, cisplatin is a classical chemotherapeutic drug. Several studies have reported its treatment effect in combination with agents targeting TAM. Researchers explored the role of PLX 3397 (PLX), an inhibitor of CSF1R, which is involved in the differentiation and recruitment of monocytes. The combination of PLX with systemically administered cisplatin provided additional benefits, causing a significant decrease in tumor burden versus monotherapy with PLX or cisplatin (98). Kawaguchi et al. proved that depletion of TAMs could inhibit lung cancer growth and enhance the anti-tumor effect of cisplatin (190). Nimesulide, dasatinib, O-ATP, A-438079 hydrochloride, A-740003, and citarinostat (ACY241) are agents that can be combined with cisplatin for therapeutic intervention in lung cancer by shifting the balance of TAMs in the TME (52, 190–192). Cui et al. developed robust NPs (MGC-GNP), which are recognizable by both cancer cells and TAMs. Pharmacodynamic assessment indicated that MGC-GNP combined with paclitaxel exhibited the highest tumor suppressive capacity compared with the other groups (193). It has been reported that TAMs with an M2 phenotype mediate resistance to gemcitabine in cancer through their influence on gemcitabine metabolic enzymes and the release of competing deoxycytidine (194). For example, Zhan et al. found that a bioactive polysaccharide isolated from Danggui Buxue Decoction improved the sensitivity of mice bearing Lewis lung carcinomas to gemcitabine by remodeling tumor-promoting M2-like macrophages into the tumor inhibitory M1 type (195). Treatment with HCQ may inhibit lung cancer by activating the anti-tumor CD8+ T cell immunity modulated by macrophages. Furthermore, this treatment increased the sensitivity of lung cancer cells to doxorubicin, suggesting that HCQ could play immune regulator and chemosensitizer roles for lung cancer chemotherapy (29). Consequently, the anti-tumor effect of chemotherapy can be elevated through combination with TAM-targeted agents. Nonetheless, additional clinical trials should be conducted to ensure the safety and effectiveness of these combinations before their extensive application to clinical practice.

### 5.3 Combination with anti-EGFR agents

Although the use of EGFR-TKIs has achieved impressive clinical response rates in patients with lung cancer, resistance to such agents is commonly observed (196). Crosstalk between cancer cells and TME plays an important role in acquired resistance to EGFR-TKI. Several studies have shown that TAMs in patients with lung cancer have detrimental effects on the clinical efficacy of EGFR-TKIs and contribute to immune evasion of EGFR-mutant cells via multiple signaling pathways (197–201). Zhang et al. also determined that, unlike M1-type TAMs, M2-type TAMs are associated with treatment response to EGFR-TKIs in patients with advanced NSCLC; hence, they may be an independent predictor of survival (202). The underlying mechanisms of TAM-induced resistance to EGFR-TKIs provide new therapeutic strategies for overcoming acquired resistance to such agents in patients with lung cancer. For example, Lin et al. reported that tumor regression triggered by osimertinib requires CD8+ T cell activation. The accumulation of TAMs in advanced tumors can inhibit the activation of CD8+ T cells and decrease the response to osimertinib. For example, the investigators found that TAM reprogramming with the systemic STING agonist MSA-2 in combination with osimertinib could reinvigorate anti-tumor immunity and lead to long-lasting tumor regression (203). An alternative mechanism also found in gefitinib-resistant lung cancer cells that reprograms TAMs through the STAT3/CD47-signal regulatory protein alpha (STAT3/CD47-SIRPα) axis has been reported. This mechanism may promote acquired resistance to EGFR-TKIs. The combination of gefitinib with a STAT3 inhibitor has the potential to attenuate acquired resistance to the latter agent. This suggests a new therapeutic strategy for the treatment of lung cancer as a result of the development of resistance to EGFR-targeted drugs (204). Recent data suggested the involvement of immune response in the anti-cancer activity of EGFR-TKIs (205). Nevertheless, the impact of tumor cell interactions with TAMs on the response to EGFR-TKI treatment is partially understood, thereby limiting the effectiveness of combination therapy. The aforementioned studies revealed a new direction for overcoming resistance to EGFR-TKIs through combination with TAM-targeted agents. However, further research is needed in this field.

### 5.4 Combination with PDT

PDT is a sophisticated treatment modality for cancer that exhibits selective killing of malignant cells via the generation of reactive oxygen species. Various PDT strategies are effective in the treatment of lung cancer, leading to improved quality of life and survival in patients with incurable malignancy (206, 207). However, as light is characterized by limited permeability and cannot penetrate bulky tumors, PDT remains underutilized in clinics (208). To overcome the obstacles of PDT, researchers have considered dual intervention on the TME and cancer cells. Thus, different strategies that combine PDT with TAM-targeted therapies have been developed. For example, cluster-induced emission luminogens (AIEgens) have been produced as novel phototherapeutic agents with excellent performance and high photostability for the induction of photodynamic and/or photothermal effects. Lin et al. designed a zwitterion-type near-infrared AIEgens C41H37N2O3S2 (termed BITT) for the treatment of lung cancer. The BITT NPs were camouflaged with TAM-specific peptide-engineered exosome membranes, which target both cancer cells and TAMs. This approach led to extensive cell death under treatment involving laser irradiation (209). The nano photosensitizer dextran sulfate (dextran sulfate-conjugated chlorin e6) is another agent manufactured to specifically target M2-type TAMs for enhancing PDT (210). Park et al. designed pH-sensitive mesoporous calcium silicate nanocomposites grafted by mannose and hyaluronic acid to target TAMs and tumor cells. Encapsulation is achieved with indocyanine green (ICG) to allow for the favorable combination of photothermal therapy and PDT (211). Similar design concepts have also been reported in numerous other studies, often involving the synthesis of novel co-operative immuno-photodynamic NPs carrying TAM-targeted agents conjugated with the photosensitizer. The goal of these designs is to effectively inhibit tumor growth and metastasis (212–214).

## 6 Outlook and conclusions

Substantial progress in understanding the biology of TAMs has been achieved in the past decade (13). In most studies, macrophages are classified into two categories, namely M1 and M2 macrophages. However, the M2 population is further sub-classified into M2a, M2b, and M2c: M2a macrophages are induced by the typical T helper 2 cytokines IL-4 and IL-13; M2b macrophages are induced by immune complexes together with TLR or the IL-1 receptor antagonist; and M2c macrophages are induced by glucocorticoids and IL-10 (215). This categorization reflects major differences in the molecular and biological behavior of tumors. Many other TAM phenotypes have been discovered based on a deeper insight into cellular immunology and improvement of analytical tools, such as single-cell RNA sequencing (64). Additional in-depth knowledge regarding the differentiation and tumor-promoting characteristics of TAMs is necessary to determine their roles in tumor progression. In general, the diversity of TAMs suggests that they perform complex functions. Firstly, the different subtypes of macrophages may affect cancer progression or suppression, potentially through the establishment of the TME, metabolic reprogramming, cancer neoangiogenesis, lymphangiogenesis, neurogenesis, and EMT formation. Secondly, TAM-related signatures may have predictive and prognostic value, and different TAMs might influence the outcome of various treatment modalities, including cytotoxic agents, ICIs, radiotherapy, and targeted therapies. Finally, macrophages may be rationally manipulated to promote an anti-cancer immune response, and such manipulations might affect outcomes in patients with tumors. For TAMs, the cell function is more important than the phenotype. Thus, the identification of TAM-associated genes which functionally participate in tumor development may help reveal critical TAM activities without the need to determine the diversity of macrophages.

The usefulness of natural products is highlighted in various strategies for the modification of TAMs, including the inhibition of macrophage recruitment, alteration of the ratio of M1/M2 TAMs, depletion of TAMs, or engineering of TAMs for drug delivery. These small molecule compounds, derived from plants, animals, minerals, and microorganisms, have shown great potential in the area of cancer therapeutics. It has been reported that several types of natural products contribute to the remodeling of the immunosuppressive TME (216). Thus, such compounds have emerged as pivotal factors that help tumors avoid recognition and attack by the human immune system and cancer immunotherapies. Furthermore, some combinations of natural products with immunotherapy have been approved by the FDA, suggesting that natural products have great potential to become qualified adjuvants for tumor immunotherapy. The selection and use of natural products that possess anti-tumor efficacy should be based on the specific type of cancer. Therefore, additional data are required to provide insight into the function of natural products in cancer therapy.

Moreover, considering the abundance of natural products, there may be other agents with anti-cancer activity that have not been discovered thus far. Researchers should focus on discovering novel and effective agents for cancer therapy that can improve global healthcare. Furthermore, a deeper and more comprehensive understanding of agents with proven ability to sensitize cancer cells to treatment is necessary. The biological differences between human and mouse macrophages also warrant investigation. Numerous molecules targeting TAMs induce tumor regression and stimulate cytotoxic responses in mouse models of tumor development. Nevertheless, the results obtained from early clinical trials in humans are less impressive. This discrepancy, to some degree, can be explained by interspecies differences between commonly used murine TAM models and human TAMs (217). Thus, the limitations of *in vitro* models in this setting should be considered to improve the design and efficacy of anti-tumor drugs targeting TAMs.

Tumor immunotherapy, particularly PD-1/PD-L1 blockade, is currently emerging as a standard therapy for lung cancer. Unfortunately, only a proportion of patients receiving anti-PD-1/PD-L1 monoclonal antibodies benefit from this treatment, as tumors in these patients develop mechanisms of immune escape. Numerous research studies revealed that TAMs induce an immunosuppressive TME and are closely related to resistance to PD-1/PD-L1 inhibitors (55, 218). Thus, combination therapy with TAM-targeted agents plus anti-PD-1/PD-L1 drugs attracted the attention of researchers. However, the correlation between PD-1/PD-L1 expression and TAMs is complex, indicating that the effectiveness of PD-1/PD-L1 inhibitors can be influenced by multiple factors. For example, PD-1/PD-L1 is expressed in both cancer cells and TAMs, and TAMs could regulate PD-L1 expression in cancer cells, further affecting tumor progression. In addition, studies demonstrated that PD-1/PD-L1 expressed in TAMs could induce TAM polarization to the M2 type. There is an urgent need to fully elucidate the underlying mechanisms through which PD-1/PD-L1 promotes the switch of TAMs to the M2-like type. The current limited understanding of the interaction between TAMs and PD-1/PD-L1 expression is a major obstacle to the advancement of TAM-targeted agents into clinical development. Given the complexity of PD-1/PD-L1 expression in the TME, it appears incorrect to recognize PD-L1 expression as the sole diagnostic marker that can guide anti-PD-1 therapy in patients with lung cancer. Hence, the identification of additional predictive and prognostic biomarkers for PD-1/PD-L1 blockade therapy in lung cancer is another direction for future research. The synergies of TAM-modification strategies and most first- and second-line treatments in lung cancer have been investigated. Nevertheless, the currently available evidence on the combination effects of other targeted therapies (except for EGFR-TKIs) is relatively limited. At present, there is ongoing research to optimize the treatment strategies for lung cancer. In conclusion, TAM plays a critical role in the progression of lung cancer, indicating that TAM-targeted therapy or combination strategies aimed at “re-educating” TAMs will emerge as a promising approach in the field of precision oncology.

## Author contributions

LL: Data curation, Writing – original draft, Writing – review & editing. GC: Formal Analysis, Writing – review & editing. SG: Software, Writing – review & editing. RH: Conceptualization, Validation, Writing – review & editing. CF: Conceptualization, Validation, Writing – review & editing.
